# Assessing Impact of Household Intervention on Indoor Air Quality and Health of Children with Asthma in the US-Mexico Border: A Pilot Study

**DOI:** 10.1155/2020/6042146

**Published:** 2020-08-03

**Authors:** Alejandro Moreno-Rangel, Juha Baek, Taehyun Roh, Xiaohui Xu, Genny Carrillo

**Affiliations:** ^1^Lancaster Institute of Contemporary Arts, Faculty of Arts and Social Science, Lancaster University, Bailrigg LA1 4YW, UK; ^2^Department of Environmental and Occupational Health, School of Public Health, Texas A&M University, 212 Adriance Lab Road, College Station, TX 77843, USA; ^3^Department of Epidemiology and Biostatistics, School of Public Health, Texas A&M University, 212 Adriance Lab Road, College Station, TX 77843, USA; ^4^Program on Asthma Research and Education, Texas A&M School of Public Health, McAllen Campus, 2102 S. McColl Road, McAllen, TX 78503, USA

## Abstract

Few studies have investigated household interventions to enhance indoor air quality (IAQ) and health outcomes in relatively low-income communities. This study aims to examine the impact of the combined intervention with asthma education and air purifier on IAQ and health outcomes in the US-Mexico border area. An intervention study conducted in McAllen, Texas, between June and November 2019 included 16 households having children with asthma. The particulate matter (PM_2.5_) levels were monitored in the bedroom, kitchen, and living room to measure the IAQ for 7 days before and after the intervention, respectively. Multiple surveys were applied to evaluate changes in children's health outcomes. The mean PM_2.5_ levels in each place were significantly improved. Overall, they significantly decreased by 1.91 *μ*g/m^3^ on average (*p* < 0.05). All surveys showed better health outcomes; particularly, quality of life for children was significantly improved (*p* < 0.05). This pilot study suggests that the combined household intervention might improve IAQ in households and health outcomes for children with asthma and reduce health disparities in low-income communities. Future large-scale studies are needed to verify the effectiveness of this household intervention to improve IAQ and asthma management.

## 1. Introduction

Asthma is a chronic disease and a widespread public health problem among children. In the United States (US), the childhood asthma prevalence was 8.4% and the rate for children's asthma attacks was 51.6% in 2017 [1]. Moreover, the rates for children's hospitalizations for Emergency Department visits were reported to be 10.7 and 74.3 per 10,000 population in 2016, respectively [1]. Children are among the populations most vulnerable to poor indoor air quality (IAQ) [2, 3]. In particular, childhood asthma rates are high among minority population residing in low-income communities and low-educated families, thereby facing environmental injustice [4]. Deprived communities are more likely to worsen existing medical conditions and live in poorer-quality environments experiencing higher air pollutant levels [5].

Exposure to air pollution is derived from both indoor and outdoor air pollutants. The health risks from exposure to indoor air pollution are known to be higher than those related to outdoor air pollution [2]. The IAQ is influenced by a mixture of pollutants from indoor (i.e., cooking, airborne suspended particles, and smoking) and outdoor (i.e., vehicular traffic and industrial) activities, as well as the building-related factors (ventilation and emissions from building materials) [3]. Biological particles, such as bacteria, fungi, and pollen, and cockroach allergens, are also associated with causing asthma or exacerbating the condition [3, 6–8]. Chemicals affecting IAQ contain carbon monoxide (CO), ozone (O_3_), radon, volatile organic compounds (VOCs), and ultrafine particulate matter (PM_2.5_). Among them, exposure to PM_2.5_ has become a major concern in public health. PM_2.5_ penetrates deeply into the respiratory barrier and enters the circulatory system causing diverse health effects, including lung cancer, cardiovascular diseases and respiratory diseases, and increased risk for asthma attacks [9–12].

Previous studies revealed that various home-based interventions, including home-based education, home environments evaluation, integrated pest management to control cockroach, and combined interventions to eliminate moisture intrusion and leaks, were effective in improving asthma conditions [13]. Particularly, a recent study presented the home-based education led by the community health workers (CHWs) improved health outcomes of children with asthma and their families in disadvantaged communities [14, 15].

Numerous studies have demonstrated that indoor air cleaning devices are a promising intervention strategy by reducing concentrations of asthma triggers in indoor air condition and bringing significant improvements in asthma symptoms in children [16, 17]. Previous studies have shown that the use of air filters reduced PM_2.5_ exposure from indoor and outdoor emissions generated from the smoking, cooking, cleaning, and other activities and led to improvements in respiratory symptoms and breathing problems for children with asthma, persistent allergic rhinitis, or bronchial hyperresponsiveness [18–22].

In addition, existing research reported that the use of air purifiers may improve IAQ and reduce asthma symptoms. A study targeting healthy college students living in the school dormitory in Shanghai, China, showed that using an air purifier resulted in a 57% reduction in PM_2.5_ concentration within operation hours and a 17% decrease of fractional exhaled nitrous oxide [23]. Another study in the Annapolis Valley, Nova Scotia, tested the air purifiers' effectiveness to remove wood smoke produced by woodstove/wood furnace, from within homes during the winter. The study revealed a 52% reduction in PM_2.5_ [24].

An intervention study at homes in Fresno, California, evaluated the effectiveness of reducing the levels of indoor air pollutants like PM_2.5_ using air purifiers to improve the health outcomes in children with asthma and allergic rhinitis. At 12 weeks, the intervention group showed the improvement in Asthma Control Test scores, whereas the control group had deterioration in the same measures [24]. Another study which examined the association of exposure to secondhand smoke (SHS) with wheezing and asthma in children showed that those exposed to SHS are 1.5 times more likely to be diagnosed with asthma or wheezing compared to unexposed children [15,25]. The other study among children with asthma aged 6–12 years old in the USA, where more than 20% of the children are exposed to SHS, demonstrated that using high-efficiency particulate air (HEPA) air filters reduced the SHS exposure, leading to an 18.5% decrease in unscheduled asthma visits [26].

However, most previous studies have focused on a single intervention tool and there is still a paucity of studies evaluating the combined household interventions to enhance IAQ and asthma-related outcomes in relatively low-income communities. Therefore, the purpose of this pilot study was to examine whether the household intervention, including both asthma education and air purifier use at home, could improve indoor air quality and health outcomes for children in the US-Mexico border area by comparing changes between pre- and postintervention.

## 2. Materials and Methods

### 2.1. Participants and Study Design

This is an intervention study conducted in McAllen, Texas, from June to November 2019, including 16 households recruited by using convenience sampling among children diagnosed with asthma and already participating in the Asthma and Healthy Homes' education study. The criteria for study participants were households: (1) having a child diagnosed with asthma aged 7 to 12 years old, (2) willing to receive asthma education, and (3) agreed to allow CHW to visit their home three times during the study, including installing air monitors and the air purifier.

**Figure 1:**
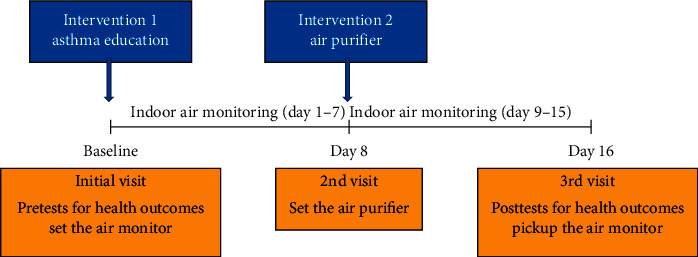
Study design of the pilot study in McAllen, Texas, from June to November 2019.

Figure 1 illustrates the study's design and plan. During the first visit (baseline), a CHW set the IAQ monitors (*Foobot*) at the child's bedroom, kitchen, and living room in each household to measure the IAQ at baseline. In addition, the CHW conducted pretests for health outcomes, including Home Environmental Personal Well-being Survey (HES), Pediatric Quality of Life Inventory Asthma Module (PedsQL), the Asthma Control Test (ACT), and Healthy Homes and Asthma Test (HHA). Then, asthma education was provided to children and their parents. A week later (Day 8), the CHW visited households to set and run the air purifier (the second intervention) in the child's bedroom. On the third visit (Day 16), the CHW conducted posttests for health outcomes using the same survey tools used in the first visit and picked up the air monitors from the children's house. The air purifier used in the study was given to participants as an incentive for their participation. Texas A&M University's Institutional Review Boards reviewed and approved the study protocol.

### 2.2. Interventions

The CHWs educated children and their parents after conducting surveys for health outcomes when they visited each household on the first visit. A holistic home-based educational intervention was provided to participants focusing on asthma control management and healthy home environments. This intervention aims to educate families on how to manage and control their child's asthma more effectively and improve their home environments, including management of the home environment and asthma symptoms, identification of common triggers, and adequate medication use and adherence. The curriculum details are explained elsewhere [15, 27]. On the second visit a week after the initial visit, the CHW installed the Levoit® Air Purifier (Model# LV-H132) in the child's bedroom where children spent the longest time in the house [28]. This air purifier has an advanced 3-stage filtration system including the prefilter, true HEPA filter, and high-efficiency activated carbon filter to capture allergens, pet hair, dander, smoke, mold, odor, and large dust particles, in addition to removing 99.97% of airborne contaminants as small as 0.3 microns as indicated by the manufacturer.

### 2.3. Indoor Air Quality Assessment

Previous studies evaluated the Foobot monitor's performance and accuracy, proving that it is a reliable tool to measure relative levels of indoor pollution [28, 29]. The Foobot was calibrated and tested in our offices in McAllen for two months before the study. The calibration equations were produced through the regression of a test conducted prior to this study. They were developed after testing and comparing the results from 10 Foobots to one kit of standard instruments (GrayWolf PC-3016A and IQ-410). The equation PM_2.5_ is as follows:

(1) $$\begin{array}{c} {\text{PM}}_{2.5}\text{GrayWolf}=0.49+0.79\left({\text{PM}}_{2.5}\text{Foobot}\right)+3.76e^{-3}\left({\text{PM}}_{2.5}{\text{Foobot}}^{2}\right). \end{array}$$

Testing of the air temperature and relative humidity sensors indicated that they are accurate, as the temperature sensor demonstrated a mean difference of −0.24°C compared to the testing instruments. Similarly, relative humidity showed a mean difference of 0.52% RH. To reduce the bias of low-cost monitors, three Foobots were used in each room, respectively, and calibration equations and data quality/corroboration, by comparison, were followed as suggested in previous studies [30,31]. The ASTM D7297-14 standard was followed to measure the physical IAQ measurements [32]. Although the IAQ is far more complex than PM_2.5_, there is more evidence about its effects to respiratory diseases, including asthma especially in children [33–35]. A series of three Foobot monitors (air temperature [−40–125°C; ±0.4°C], relative humidity [0–100% RH; ±4% RH], and PM_2.5_ [0–1,300 *μ*g/m^3^; ±4 *μ*g/m^3^]) were installed at the bedroom, kitchen, and living room in each household. During five-minute intervals, the Foobot air monitors collected data about air quality parameters over two weeks in each household. In particular, PM_2.5_ was measured continuously throughout the study period and was estimated as *μ*g/m^3^ [36]. Data was saved automatically in secured online storage and stored in an encrypted computer safely.

### 2.4. Health Outcomes Assessment

The research team applied the four survey tools, including HES, ACT, PedsQL, and HHA, to assess health outcomes before and after the intervention. First, the HES is a tool to assess general well-being about health conditions consisting of eight questions (total score: 8), such as dry eyes, runny nose, headache, and dry, itching, or irritated skin with a higher score denoting more symptoms [30]. Second, the ACT encompasses seven self-assessment questions for asthma control (total score: 27) to determine if child's asthma symptoms are well controlled, including general asthma symptoms (cough, wheezing, and sleep disturbance), the frequencies of asthma symptoms, and the effect of asthma on daily functioning. Higher scores indicate better asthma control [30].

Third, the PedsQL Asthma Module is a modular instrument designed to measure asthma-specific health-related quality of life in children aged 2–18 years old [33]. This survey tool includes 28 items in subsections of asthma symptoms, treatment problems, worry, and communication problems. Each item used a 4-point response scale with five categories: 0 (never), 1 (almost never), 2 (sometimes), 3 (often), and 4 (almost always). The total score ranged between 0 and 112. Lastly, the HHA test includes 10 questions (total score: 10) to evaluate knowledge of asthma symptoms and management, triggers, and environmental and behavioral risk factors [34]. Overall, the ACT test was applied to children, and the PedsQL, HES, and HHA were asked to parents. The lower score means an improvement in the HES and PedsQL test, while the higher score means an improvement in the ACT and HHA test.

### 2.5. Statistical Analysis

Among 16 households who participated in this study, 13 households were included in the analysis. Three households were excluded in this study since significant amounts of air monitoring data were lost. Descriptive statistics were calculated to estimate mean and standard deviation (SD) for temperature and relative humidity. The PM2.5 concentrations were right-skewed and geometric means were calculated after log transforming them to compare the change between pre- and postintervention. The *t*-tests were conducted to compare the PM_2.5_ levels for each household between pre- and postintervention. The Wilcoxon signed-rank tests were performed to compare overall changes in the PM_2.5_ levels and the overall scores from four different survey tests among all participants between pre- and postintervention, respectively. A *p* value of less than 0.05 was considered statistically significant. All statistical analyses were conducted by using SAS version 9.4 (Cary, NC).

## 3. Results

Table 1 shows the demographic and house characteristics of 13 participating households. They included seven boys and six girls aged 7 to 12 years old (average: 9.5 years old). Six participants (46.2%) had pets at their homes and seven families (53.8%) used electronic stove. The number of people living in the house was 5.6 on average range from 3 to 10. In terms of house characteristics, six households (46.2%) had tile floors, six (46.2%) reported hardwood floors, and one (7.6%) had carpet floor. In addition, most of the households had an open-plan (69.2%) and kitchen range (69.2%). As instructed during the asthma education, almost half of the households ventilated (opened their windows and doors always during the day), the other half ventilated their home sometimes, but only one did not ventilate. The indoor average air temperature and relative humidity measured from 13 participants' homes for two weeks were 25.8°C (range: 22.5∼29.8) and 50.7% (range: 41.3∼56.7), respectively.

**Figure 2:**
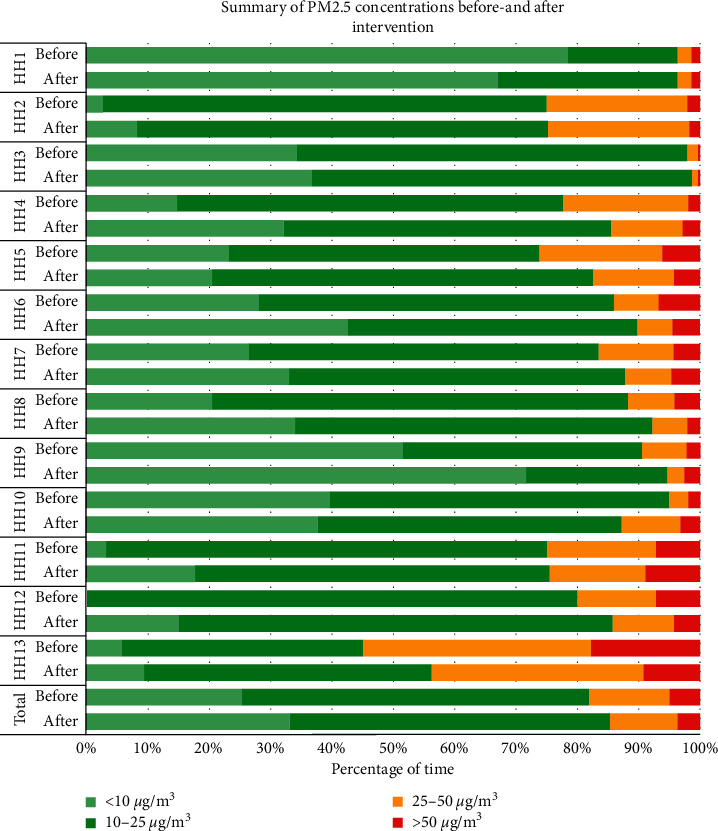
Summary of PM_2.5_ concentrations in pre- and postintervention periods in each household.

Table 2 describes the results of geometric means of PM_2.5_ concentration levels between pre- and postintervention for each household. In all of the three places, the mean PM_2.5_ level significantly decreased in 13 households on average by 1.91 *μ*g/m^3^ (*p* < 0.05). In particular, eight households (61.5%) showed a significant decrease in the PM_2.5_ level. The most significant PM_2.5_ mean difference between pre- and postintervention among 13 participants was in the bedroom (-2.13 *μ*g/m^3^), where the air purifier was installed and children spent the longest time at home. The mean PM_2.5_ levels in HHs 4, 6, 8, 9, 12, and 13 showed consistently significant improvements in all locations within the house. The PM_2.5_ levels in HHs 2 and 3 were shown to decrease significantly only in the kitchen.  Figure 2 illustrates changes in PM_2.5_ concentrations before and after the intervention for each household visually. It indicates that most of the households had improvements in PM_2.5_ levels after the intervention.

In addition, we conducted four different tests to examine the change of health conditions for children with asthma and their parents between pre- and postintervention. The results of comparing children's health outcomes and their parents' knowledge regarding asthma between pre- and postintervention for each household were shown in Table 3. We found that all tests displayed improvements in outcomes for children and their parents. Particularly, the average scores of HES and PedsQL tests decreased by 0.15 and 6.08, respectively. The mean scores of ACT and HHA tests increased by 0.62 and 0.23, individually. However, only the difference in the PedsQL test was statistically significant (*p* < 0.05), and the results from the other three tests were not statistically significant. In addition, we estimated the average outdoor PM_2.5_ and ozone level using the EPA's Outdoor Air Quality Data to compare the outdoor air quality during the study periods before and after implementing air purifier and no difference was found in outdoor air quality in most households (Table 4) [37], excluding the influence of outdoor air quality on the improvement in indoor air pollution or health outcomes.

## 4. Discussion

Asthma is a chronic disease that adversely affects the overall quality of life for children during their formative years, and their exposure to indoor air pollutants may exacerbate their asthma. However, effective strategies are available to reduce exposure and prevent asthma symptoms [29]. In this pilot intervention study, the effectiveness of the combined intervention with asthma education and air purifier was investigated using a crossover design for 15 days. Asthma education was provided at baseline and a low-cost affordable air purifier was operated for a week after a week of air monitor optimization. Our study showed a statistically significant decrease in the mean PM_2.5_ concentration by 1.91 *μ*g/m^3^ and the PedsQL score by 6.08 points, indicating the improvement of IAQ and quality of life in children with asthma.

Previous studies have shown that families educated with a curriculum based on Asthma and Healthy Homes had improved asthma symptoms and increased knowledge of children and their parents as well as the quality of life for the family [14, 15, 28]. The curriculum is focused on a holistic educational intervention including the signs and symptoms of asthma, the disease management, common triggers of asthma, adequate use of asthma medications, emergency action plans like an asthma attack, and components of an asthma action plan [15]. It also included the Seven Principles of Healthy Homes, developed by the National Healthy Homes Training Center and Network. The educational components focused on how to keep a home dry, clean, ventilated, pest-free, safe, contaminant-free, improving the indoor environment, and decreasing hazardous exposures within the home [15].

In contrast to most previous studies that applied a single type of intervention, this study implemented a combined household intervention that included air purification and asthma education and evaluated changes in asthma for children in this study. We found that most of the households showed the PM_2.5_ level decreased after the combined intervention, indicating the consistent results as previous studies. Specifically, the frequency of detecting PM_2.5_ levels below the World Health Organization (WHO) annual target of 10 *μ*g/m^3^ was increased, and subsequent improvements in children's asthma control and management were demonstrated by four different surveys, regardless of the difference in some household characteristics [38]. This result is consistent with the findings of other intervention studies using HEPA filtration. A study found that indoor PM_2.5_ level was reduced from 7.6 *μ*g/m^3^ to 3.4 *μ*g/m^3^ after the HEPA intervention [39], and another study also reported a significant reduction in PM_2.5_ level from 8.0 *μ*g/m^3^ to 4.8 *μ*g/m^3^ with the use of HEPA [40].

As awareness of the impact of indoor air pollution on health increases, new monitoring technologies are developed to monitor the quality of indoor air. While international standards such as ISO 16000-1 [41] and the EPA standard protocol for characterizing IAQ [42] establish routines for IAQ monitoring, the desire to simultaneously monitor different rooms, initial investment, and need for trained personnel to handle and analyze the data pose clear challenges. Additionally, in alignment with the aim of this work, low-cost monitors were considered a suitable option. However, their use may have inconveniences, i.e., additional data quality protocols and the risk of data loss if their connection is lost [43]. Another critical consideration is the use of a variety of algorithms used by different manufacturers to convert the sensor output into a concentration value for each pollutant [44]. There is, however, an important limitation of the Foobot. It is not suitable for outdoor use, limiting the collection of outdoor data and the limited air pollutants it measures. Despite the low-cost monitors' limitations, there are also some benefits: advanced software processing of air quality data, compact size, continuous measurements, potential of customization, deployment, high scalability, low maintenance, low-cost, low-power consumption, possible auto-calibration, and quick responses [25].

Our study has several limitations. Firstly, the total study period, including pre- and postintervention periods, was 15 days so that it might be a shorter time to see substantial changes in indoor air condition and participants' health outcomes when compared with other similar studies that had at least 4 weeks [40, 45]. However, we observed a 12% significant reduction in the mean PM_2.5_ level and some positive outcomes in children with asthma, providing a promising basis for future studies. Second, some of the air quality data collected from air monitors were lost due to poor Internet connection in the study field even though a strong data collection and management plan was used in this study. Since some houses were located in remote sites, where the Internet connection was not stable, we could not store the data on our servers and the chosen instrument did not have internal data storage. However, the PM_2.5_ levels were measured every five minutes for two weeks, and enough number of measurements was obtained to compare air quality before and after the intervention except three households that were excluded.

Third, we did not use a traditional air monitoring approach to collect samples and analyze later with highly accurate and precise analytical instruments, such as gravimetric sensor, because they require high cost and skills to operate and maintain, leading to the relatively short duration of measurements [28, 46]. Instead, we opted for longer monitoring periods using low-cost and direct-reading consumer monitor (*Foobot*), which can be accessible to the families in low socioeconomic status and viable for large-scale health programs. This instrument is widely used to monitor indoor air quality, and its performance and quality have been demonstrated in the previous studies [28, 47, 48].

Fourth, due to the small sample size, we could not use the statistical model to adjust the characteristics of socioeconomic status, which may affect asthma severity. Given that similar studies included more than 40 participants [39, 40, 45], the future study should have more sample sizes to obtain better results by conducting a statistical analysis. However, the socioeconomic status of participating families in this study is homogeneous as low-education and low-income Hispanic population supported by public health insurance aid was recruited. Lastly, like any other similar study, participants' engagement was difficult to retain. We had difficulties in setting up appointments with participating households, including last-minute cancellations, and using the air purifier. Although air purifiers were intended to stay turned on during the entire study period, some households turned them off when the child was not at home to save energy.

Despite some limitations, there are several strengths to this study. First, we examined the effect of the combined household intervention, including the use of affordable air purifier and asthma education, on health outcomes of children with asthma in families with low socioeconomic status, demonstrating a more effective way to improve pediatric asthma population's health outcomes and reduce health disparities in low-income communities. Second, validated air monitors were installed in three different places of each household to measure overall air condition in households more correctly and eliminate any potential bias caused by the instruments. Third, we used multiple survey tools to measure various health outcomes of children comprehensively before and after the intervention. Finally, our study proposed a potential intervention method to reduce the risk of other respiratory diseases, lung cancer, and cardiovascular diseases related to PM_2.5_ exposure [49].

## 5. Conclusions

This study evaluates the effects of a combination of an air purifier and asthma education as a household intervention on IAQ and children with asthma health outcomes in a low-income community. This pilot study suggested that using the combination of asthma education and an air purifier at home might enhance IAQ, contributing to improving children's health outcomes and reducing health disparities. Future large-scale studies are needed to verify the effectiveness of household intervention to enhance IAQ and asthma management. Also, further research should include a comparison between an intervention group and a control group to observe the impacts of using only asthma education or IAQ management or both to children with asthma health conditions.

## Figures and Tables

**Table 1:** Demographic and household characteristics of participants (*N* = 13).

HH	Gender	Age (years)	Pets	Stove	Number of people living in the house	Floor	Open concept house^a^	Kitchen range	Ventilation^b^	Temperature (°C) (SD)	Relative humidity (%) (SD)
1	Boy	7	Yes	Gas	10	Tile	No	Yes	Sometimes	25.0 (3.5)	52.9 (4.7)
2	Girl	8	Yes	Gas	7	Tile	Yes	No	Always	28.8 (3.4)	54.2 (5.7)
3	Girl	10	No	Electronic	5	Tile	No	Yes	Sometimes	25.7 (0.8)	48.3 (1.7)
4	Boy	10	No	Electronic	6	Hardwood	Yes	Yes	Always	29.8 (4.5)	51.3 (7.4)
5	Girl	10	Yes	Gas	5	Hardwood	Yes	Yes	Always	29.8 (4.6)	53.1 (5.4)
6	Boy	12	No	Electronic	4	Hardwood	Yes	No	Always	28.5 (2.2)	54.1 (4.2)
7	Girl	9	Yes	Gas	5	Hardwood	Yes	No	Always	24.2 (2.0)	46.6 (3.2)
8	Boy	11	No	Electronic	5	Tile	No	Yes	Sometimes	26.0 (0.8)	46.2 (4.4)
9	Girl	9	Yes	Gas	4	Tile	Yes	Yes	Always	24.4 (1.1)	45.2 (2.6)
10	Boy	8	No	Electronic	6	Carpet	No	Yes	Never	22.5 (1.5)	56.7 (4.6)
11	Girl	8	No	Electronic	7	Hardwood	Yes	No	Sometimes	22.7 (3.0)	47.2 (5.1)
12	Boy	10	Yes	Gas	3	Tile	Yes	Yes	Sometimes	25.0 (1.9)	48.2 (6.4)
13	Boy	12	No	Electronic	6	Hardwood	Yes	Yes	Sometimes	22.5 (2.9)	54.6 (6.9)

*Excluded participants due to missing data for indoor air quality (N* *=* *3)*											
	Boy	6	Yes	Gas	5	Tile	No	Yes	Sometimes	25.7 (1.7)	49.1 (3.7)
	Boy	8	Yes	Gas	8	Tile	Yes	Yes	Never	24.0 (0.8)	52.3 (2.1)
	Boy	8	Yes	Electronic	6	Hardwood	Yes	Yes	Always	20.5 (1.8)	53.3 (4.2)

Abbreviation: HH, household; SD, standard deviation. ^a^Open concept house includes kitchen and living room in one single area with no division. ^b^Always, windows and doors always open; sometimes, open windows once/twice a month; never, does not ventilate home. ^c^Average.

**Table 2:** Geometric means of PM_2.5_ concentrations between pre- and postintervention for each household.

HH	Total			Bedroom			Kitchen			Living room		
	Pre	Post	Difference	Pre	Post	Difference	Pre	Post	Difference	Pre	Post	Difference
1	7.91	10.48	2.57	8.61	9.63	1.03	9.61	11.28	1.67	5.51	10.53	5.02
2	19.49	18.38	−1.11^*∗*^	15.36	17.17	1.81	20.11	15.00	−5.11^*∗*^	23.00	22.98	−0.02
3	11.08	10.74	−0.34	12.35	12.26	−0.09	13.23	12.35	−0.88^*∗*^	7367	7.62	−0.05
4	19.46	13.27	−6.19^*∗*^	13.76	11.59	−2.17^*∗*^	21.99	15.67	−6.32^*∗*^	22.62	12.56	−10.06^*∗*^
5	16.39	15.96	−0.43	20.28	17.44	−2.84^*∗*^	15.20	17.69	2.49	13.69	12.76	−0.93^*∗*^
6	15.50	12.81	−2.69^*∗*^	11.00	9.37	−1.63^*∗*^	19.22	16.62	−2.60^*∗*^	16.27	12.45	−3.82^*∗*^
7	14.41	13.50	−0.91	18.00	13.28	−4.72^*∗*^	6.34	7.91	1.57	18.89	19.30	0.41
8	15.19	12.16	−3.03^*∗*^	17.08	13.59	−3.49^*∗*^	16.73	14.22	−2.51^*∗*^	11.77	8.68	−3.09^*∗*^
9	11.58	9.75	−1.83	10.72	7.54	−3.18^*∗*^	12.10	11.47	−0.63^*∗*^	11.93	10.25	−1.68^*∗*^
10	11.25	12.73	1.48	8.90	9.58	0.68	13.96	15.82	1.86	10.89	12.78	1.89
11	19.82	18.34	−1.48^*∗*^	18.21	18.58	0.37	22.34	18.84	−3.50^*∗*^	18.91	17.59	−1.32^*∗*^
12	22.07	16.67	−5.40^*∗*^	23.08	16.25	−6.83^*∗*^	24.50	20.90	−3.60^*∗*^	18.62	12.87	−5.75^*∗*^
13	20.05	23.52	−5.53^*∗*^	35.39	28.81	−6.58^*∗*^	23.41	16.66	−6.75^*∗*^	28.34	25.10	−3.24^*∗*^
Total	16.40	14.48	−1.91^*∗*^	16.36	14.24	−2.13^*∗*^	16.83	14.96	−1.87^*∗*^	16.01	14.27	−1.74^*∗*^

*Note.* The two-sample *t*-test and Wilcoxon signed-rank test were used to test the improvement within each household and over 13 households between pre- and postintervention, respectively. ^*∗*^Significant at *p* < 0.05

**Table 3:** Changes in scores^a^ for children's health outcomes and parent's knowledge of asthma between pre- and postintervention for each household by the survey.

HH	HES (total score: 8)		ACT (total score: 27)		PedsQL (total score: 112)		HHA (total score: 10)	
	Pre	Post	Pre	Post	Pre	Post	Pre	Post
1	1	0	22	24	15	29	7	8
2	6	5	24	24	23	13	9	8
3	4	3	26	25	9	9	10	8
4	2	4	22	22	31	27	8	8
5	5	7	19	23	15	0	5	8
6	0	1	27	25	14	23	6	8
7	7	3	23	23	16	19	9	10
8	2	0	27	27	23	9	7	8
9	3	1	23	21	48	20	10	10
10	0	2	20	25	15	0	8	10
11	3	5	27	26	13	5	8	6
12	7	7	25	26	22	10	10	7
13	2	2	25	27	6	7	7	8
Total	3.23	3.08	23.8	24.5	19.2	13.2	8.00	8.23
Difference	−0.15		0.62		−6.08^*∗*^		0.23	

Abbreviation: HES, Home Environmental Personal Well-being Survey; ACT, Childhood Asthma Control Test; PedsQL, Pediatric Quality of Life Inventory Asthma Module; HHA, Healthy Homes and Asthma Test. *Note.* The Wilcoxon signed-rank test was used to test the overall improvement across 13 households between pre- and postintervention. ^a^ The lower score means improvement in the HES and PedsQL test, while the higher score means an improvement in the ACT and HHA test.; ^*∗*^Significant at *p* < 0.05.

**Table 4:** Outdoor PM_2.5_ and ozone levels in McAllen^a^ during the study period for each household.

HH	PM_2.5_ (*μ*g/m^3^)				Ozone (ppm)			
	Days 1–7		Days 9–15		Days 1–7		Days 9–15	
1	10.3	(3.6)	11.1	(3.4)	0.028	(0.009)	0.029	(0.011)
2	10.4	(3.5)	10.5	(3.9)	0.034	(0.012)	0.025	(0.005)
3	13.6	(2.2)	12.8	(4.5)	0.020	(0.003)	0.026	(0.003)^b^
4	13.6	(2.2)	12.8	(4.5)	0.020	(0.003)	0.026	(0.003)^b^
5	11.8	(4.4)	13.9	(3.1)	0.020	(0.003)	0.025	(0.004)^b^
6	6.0	(1.1)	7.6	(3.4)	0.034	(0.008)	0.029	(0.004)
7	7.0	(2.3)	6.2	(1.6)	0.032	(0.009)	0.030	(0.004)
8	8.4	(3.4)	6.1	(1.7)	0.033	(0.009)	0.030	(0.004)
9	8.4	(3.4)	6.1	(1.7)	0.033	(0.009)	0.030	(0.004)
10	5.8	(2.0)	7.3	(2.0)	0.033	(0.008)	0.033	(0.009)
11	5.3	(1.6)	7.1	(2.0)	0.030	(0.008)	0.035	(0.009)
12	5.3	(1.6)	7.1	(2.0)	0.030	(0.008)	0.035	(0.009)
13	6.9	(1.5)	4.8	(1.8)	0.031	(0.009)	0.037	(0.005)

Source: Outdoor Air Quality Data, US Environmental Protection Agency (https://www.epa.gov/outdoor-air-quality-data/download-daily-data). *Note.* The two-sample *t*-test was used to test the difference of concentrations between Days 1–7 and Days 9–15 within each household. Expressed in mean of daily measurements (SD); ^a^ McAllen-Edinburg-Mission, TX. ^b^ Significant at *p* < 0.05.

## Data Availability

The data used to support the findings of this study are available from the corresponding author upon request.
